# Supplementary material for: Development and validation of an artificial intelligence model based on liver CSE-MRI fat maps for predicting dyslipidemia
Source: PLOS Digit Health. 2026 Jan 7;5(1):e0001119. doi: 10.1371/journal.pdig.0001119 (PMC12779152; doi:10.1371/journal.pdig.0001119)
Supplement: Attachment — Submitted filename: Response to Reviewers.docx [file pdig.0001119.s002.docx]

**Dear Editor,**

**We would like to express our gratitude to you and the reviewers for the thorough evaluation of our manuscript titled "Development and validation of an artificial intelligence model based on liver CSE-MRI fat map for predicting dyslipidemia." We appreciate the insightful comments and constructive feedback, which have greatly contributed to improving the quality of our work. Below, we provide detailed responses to each of the reviewers' comments and suggestions.**

Reviewer #1: Comments

1. Sample Size and Generalizability

• The study includes only 89 patients, which raises concerns about how well the findings can be generalized to a broader population.

• The class imbalance (e.g., significantly more cases with high TG than normal TG) could affect the model’s ability to perform consistently across different lipid levels.

Response: Your concern is very valid. We acknowledge that the model derived from 1,757 slices from 89 patients cannot be directly generalized for wider application. However, this is a valuable attempt, as currently, no other studies have tried to use MRI fat quantification data to directly predict lipid indicators. On the other hand, while we only have 89 patients, obtaining this data from clinical sources has been quite challenging. Regarding the class imbalance, during the early stages of experimental design, we attempted methods such as data augmentation, oversampling, and weighted cross-entropy loss, but these quickly led to model overfitting. Conversely, L2 regularization, a smaller learning rate, and batch size contributed positively, as mentioned in the methods and discussion sections. Additionally, the current data imbalance reflects the real-world situation. For example, in clinical settings where dyslipidemia is suspected, there are indeed more cases of TG abnormalities than normal TG levels. Therefore, in practical applications, the model would handle more images with TG abnormalities. We believe that data processing techniques alone may not resolve the issue of applying the model directly; expanding the dataset is crucial.

2. Model Selection and Justification

• The manuscript tests several AI models and selects ResNet18 as the best-performing option. However, the reasoning behind this choice is not fully explained.

Response: In terms of model selection, we first determined the type and size of the pre-trained network we needed. We then reviewed literature from the past five years to select five models that are frequently used and have high accuracy in medical image classification,This is the rationale behind choosing these five models. In the revised manuscript, we further elaborated on why ResNet18 was chosen and analyzed the reasons for its outstanding performance.

3. Performance of the Model (Particularly for TC)

• The F1-score for total cholesterol (TC) is quite low (0.571), suggesting the model struggles to predict this parameter accurately.

• Additionally, confidence intervals for AUC and accuracy values are missing, which are essential for assessing the robustness of the model.

Response: You are correct. We acknowledge that the prediction performance for TC among the various lipid indicators is the poorest and is essentially unusable. This is thoroughly mentioned in the article, and we expanded on the primary reasons in the discussion section. We believe that besides data issues, the fundamental reason is that total cholesterol (TC) includes cholesterol from LDL-C, HDL-C, and VLDL-C. Patients with hyperlipidemia often exhibit low LDL-C and high HDL-C, and since MRI signals are indirect reflections of water and fat components, analyzing a single component is already challenging, let alone mixed total cholesterol (TC). Thus, we believe TC has very complex features, and when the training sample size is small, it is difficult for the computer to find effective features that capture its variability. We have provided explanations and analyses in the discussion section to help readers objectively understand the technical limitations rather than solely pursue high accuracy. We have also added confidence intervals and other statistical information in the text.

4. Clinical Relevance and Validation

• While the model’s results are interesting, the lack of an external validation dataset is a significant limitation.

Response: You are correct, but this might be a misunderstanding due to the wording. We have added modifications and explanations in the text. Figure 2-1 provides a detailed interpretation of the data division, where the training set includes a training subset and a validation subset (for cross-validation), and the test set is an external dataset that is not involved in training. The patients in the test set were reserved in advance, and different images from the same patient do not appear in both the test set and the training set. On the other hand, we agree with your view. Our experiments are still in the exploratory stage, and more samples and validation in larger independent cohorts are needed before clinical application.

Reviewer #2:

1. The authors have responded with "ok" to the competing interests and data availability statements, which is insufficient. Please explicitly disclose any competing interests, if applicable, or state that none exist. Additionally, provide a clear and detailed data availability statement, specifying whether the data are publicly available, subject to restrictions, or not accessible, along with relevant repository links or access conditions.

Response: We have expanded the statement of competing interests as per your suggestion.

2. Throughout the manuscript, the authors use `accuracy_score`, `f1_score`, `precision_score`, and `recall_score`. Please use the standard metric names (accuracy, F1 score, precision, recall) instead. In the abstract, simply refer to them as "accuracy metrics" rather than listing each one, as an exhaustive list is unnecessary.

Response: In the abstract, we changed terms like accuracy_score, f1_score, Precision_score, and recall_score to accuracy metrics.

3. Abstract Results: Mentioning transfer learning again in results is redundant. Perhaps, rephrase the first sentence. The authors could improve clarity and readability by restructuring the last sentence.

Response: We have rephrased the text according to your suggestion.

4. Introduction - Paragraph 1: Please provide a clear definition of dyslipidemia to enhance readability for a non-clinical audience.

Response: We have expanded the opening section of the introduction as per your suggestion.

5. Introduction - Paragraph 2: The last few lines lack proper citations. The authors should provide appropriate references to support the statements.

Response: We have added references as per your suggestion.

6. Introduction - Paragraph 3: The phrase "with the increasing demand for medical experience" seems a bit vague. It might be clearer to specify the increasing demand for "improved healthcare" or "more efficient healthcare services." "But it can not" should also be changed to "however, it cannot."

Response: We have rephrased the text according to your suggestion.

7. Introduction - Paragraph 3: The gap statement at the beginning is very vague, the authors should make it more objective and concrete.

Response: We have rephrased the text according to your suggestion.

8. Introduction - Paragraph 4: The first two lines are redundant.

Response: The first two lines of this paragraph are intended to help medical readers understand and accept the AI work we have done by providing sufficient basis and sources.

9. Methods: The Experimental Design and Model Training sections provide overlapping information. It would be more efficient to remove the Experimental Design section and restructure the Methods section to begin with the Dataset Description, followed by a discussion of Model Development and Statistical Evaluation.

Response: We have deleted the "Experimental Design" section and restructured other sections as per your suggestion.

10. Methods - CSEMRI Scanning: In the imaging parameters, it would be helpful to include the number of slices for each MRI scan, as well as the slice thickness.

Response: We have added descriptions as per your suggestion.

11. Methods: Repeatedly mentioning the libraries used throughout the manuscript is unnecessary, particularly for common ones like 'os' and 'glob.' It would be more efficient to make the code publicly available, which would allow readers to easily access the full list of libraries and dependencies.

Response: We have modified the descriptions as per your suggestion.

12. Methods - Image Classification and Preprocessing: A total of 1,757 images were extracted from 89 MRIs, which were then split into training and test sets. It is unclear whether this split was random or done along patient lines (e.g., 19 MRIs in the test set and 70 in the training set, with images extracted accordingly). If the split was random, this could potentially lead to data leakage. The authors should explicitly clarify the method of data splitting. If the split was random, I recommend reseparating the data along patient lines and retraining the models to avoid potential bias.

Response: You are correct, and this is an issue we have considered. We have added explanations in the text. The test set is an external dataset that is not involved in training, and the patients in the test set were reserved in advance. Different images from the same patient do not appear in both the test set and the training set, which is what you described as separating the data along patient lines and then counting the number of images in the test set. This also explains why the number of test set images for each lipid indicator is different, as each patient has a different number of images.

13. Methods - Model Training: The authors mention '... and other lightweight medical image classification networks,' but the figures and tables only present the five architectures discussed in the manuscript. It would be helpful to clarify whether additional architectures were considered or to update the manuscript to reflect only the five mentioned.

Response: You are correct. In terms of model selection, we first determined the type and size of the pre-trained network we needed. We then reviewed literature from the past five years to select five models that are frequently used and have high accuracy in medical image classification. This is the rationale behind choosing these five models. Other lightweight models were eliminated during the literature screening and did not participate in modeling experiments. We have added explanations in the revised manuscript, and we further elaborated on why ResNet18 was chosen and analyzed the reasons for its outstanding performance.

14. Methods - Model Evaluation and Statistical Analysis: The details on how cross-validation works are unnecessary. This section lacks statistical rigor—was any statistical comparison performed between the models' performances to identify the best-performing model? Including such analysis would strengthen the manuscript.

Response: We have removed the details on the principles of cross-validation and added statistical analysis of differences as per your suggestion.

15. Results: The Results section is lacking in detail and mainly repeats information from the Methods section. It needs to be rewritten to provide a more in-depth analysis and clear presentation of the findings.

Response: We have rewritten the Results section according to your suggestion.

16. Discussion: The entire Discussion section is highly redundant, with a substantial portion repeating information from the Methods section. The authors should focus on discussing the results in depth and emphasize the clinical applicability of their findings.

Response: We have rewritten the Discussion section according to your suggestion.

17. Figures 2 and 3 convey the same information. It would be more efficient to merge them into a single figure to avoid redundancy.

Response: We have merged the figures according to your suggestion.

18. Figure 4 has several issues: there is a lot of overlapping values, the legend is repeated four times, the y-axis scale is inconsistent across plots, and the alignment is not optimal. Additionally, while the authors mention calculating the standard deviation, error bars are not presented in the figure.

Response: We have reformatted the figure as per your suggestion.

19. The fonts in Figure 5 are too small, and there are no subplot titles.

Response: We have added subplot titles to the figure as per your suggestion.

20. No standard deviations are provided in any of the results tables. I also suggest that the authors include a comprehensive table in the supplementary materials with all metrics for the models' cross-validation results.

Response: We have added standard deviation and statistical difference data as per your suggestion.

**We hope that our revisions and responses have adequately addressed the reviewers' concerns and improved the manuscript. We are committed to further enhancing the quality of our work and are open to any additional suggestions. Thank you once again for the opportunity to revise our manuscript. We look forward to your favorable consideration for publication.**

**Sincerely,**

**JIANG Bo**

**Department of Radiology, The Second Xiangya Hospital of Central South University**

**jiangbo@csu.edu.cn**
