# Supplementary material for: Development and validation of an artificial intelligence model based on liver CSE-MRI fat maps for predicting dyslipidemia
Source: PLOS Digit Health. 2026 Jan 7;5(1):e0001119. doi: 10.1371/journal.pdig.0001119 (PMC12779152; doi:10.1371/journal.pdig.0001119)
Supplement: Attachment — Submitted filename: Response to Reviewers2.docx [file pdig.0001119.s003.docx]

**Dear Editor,**

**We would like to express our gratitude to you and the reviewers for the thorough evaluation of our manuscript titled "Development and validation of an artificial intelligence model based on liver CSE-MRI fat map for predicting dyslipidemia." We appreciate the insightful comments and constructive feedback, which have greatly contributed to improving the quality of our work. Below, we provide detailed responses to each of the reviewers' comments and suggestions.**

**Reviewer #2:**

**1. One of the major concerns is the clinical relevance of this technology. It would be infeasible for patients to be subjected to expensive MR imaging solely to predict dyslipidemia risk. A definitive diagnosis of dyslipidemia would still be most effectively/efficiently done via blood draw since MR for something as common as dyslipidemia would be prohibitively expensive for most primary care providers to consider and would take away imaging time from acute patients. This would seem to be more of an incidental finding for when an abdominal MRI is clinically indicated for another complaint.**

**Response: We sincerely thank the reviewer for raising this critical point regarding clinical relevance and cost-effectiveness. We fully agree that performing MRI solely for dyslipidemia screening is neither practical nor economical compared to standard blood tests. In light of this feedback, we have significantly revised the discussion section (particularly the "Clinical Significance" subsection and "Conclusion") to clarify the intended application scenarios of our AI model. We now explicitly state that our approach is not proposed as a primary screening tool, but as a method to add value using existing imaging data. Specifically, when patients undergo abdominal MRI for other reasons (e.g., assessment of fatty liver, liver focal lesions, or other clinical indications) and the CSE-MRI sequence is included, the acquired fat map can be opportunistically analyzed by our AI model without adding any extra scan time or cost, thereby providing an incidental prediction of dyslipidemia risk as an early warning signal and facilitating timely referral for confirmatory blood tests. We believe this "opportunistic screening" model enhances the utility of routinely collected clinical data. On the other hand, in my country, the cost of a liver MRI scan has significantly decreased, currently costing only 430 RMB (60 USD), and AI-assisted diagnosis can be billed separately at 60 RMB (8.4 USD), which can predict multiple lipid indices comparable to the cost of blood tests.**

**2. The Results section lacks sufficient quantitative and statistical detail. Multiple performance claims are made without reporting actual values (e.g., average accuracy, F1-score, PPV, TPR, AUC, etc.). While Tables 1 and 2 are referenced, key numerical findings should also be summarized in the text.**

**Response:We appreciate this valuable suggestion from the reviewer. We agree that integrating key quantitative results into the main text can improve readability and impact. We have thoroughly revised the Results section to directly incorporate fundamental numerical findings into the narrative. For instance, we now explicitly report the average accuracy (ACC) and loss function values during 8-fold cross-validation with the ResNet18 model. Additionally, when describing the optimal model's performance on the independent test set, we have directly written key performance metrics (F1-Score, ACC) from Table 3 into the text. This modification enables readers to immediately grasp the model's performance without solely relying on tables.**

**3. The narrative flow of the Results and Discussion sections is weak. The transition from model comparison to model selection and final evaluation is abrupt and lacks justification. The rationale for selecting ResNet18 over other models needs to be more comprehensive i.e., was this decision based solely on accuracy, or were other factors such as variance or training time considered? Additionally, Figure 3 should include appropriate error bars to support the comparison, and the narrative should be revised for clearer structure.**

**Response:We appreciate the reviewer's feedback on improving narrative flow, justification, and Figure 3. We have made the following modifications:**

**(1) We reorganized the Results section to directly incorporate fundamental numerical findings into the narrative, enabling readers to immediately grasp the model's performance without solely relying on tables.**

**(2) We expanded the Discussion section using a progressive approach, starting with Clinical Significance and then discussing Model Design and Tuning, detailing the initial choice of ResNet18 and other comparative models. In the Model Effectiveness Analysis section, we elaborated on multiple arguments for choosing ResNet18 (accuracy, generalization balance, residual architecture advantages). Finally, we discussed Future Work and Prospects.**

**(3) We updated Figure 3 by adding error bars (representing standard deviation) for each model and metric mean, providing a visual representation of model performance and stability.**

**4. The choice of training for 40 epochs is not justified. Did the authors consider or implement early stopping to prevent overfitting?**

**Response:This is a reasonable concern. We have revised the "Model Training" subsection in the Methods section to provide justification for the number of epochs and clarify our strategy to prevent overfitting. We now state that the number of epochs (40) was chosen based on preliminary experimental experience, which indicated that model performance on the validation set typically stabilized or began to decline after this point. More importantly, we explicitly describe our strategy: "Although a fixed number of epochs was set, we saved the model state after each epoch. The model selected for evaluation was not necessarily the final epoch model but the one that achieved the lowest loss and highest accuracy on the internal validation set during the entire training process. This approach functionally resembles early stopping but without a patience value, aiding in broadly selecting models with minimal overfitting potential."**

**5. Citations for biomarker cutoff values are missing. These should be clarified in the Methods section.**

**Response:We thank the reviewer for pointing out this omission. We have revised the "Image Classification and Preprocessing" section to clearly state that the cutoff values reference the latest guidelines proposed by the Chinese [9] for ASCVD low-risk population screening (all samples are from China) and have listed the specific cutoff values.**

**6. Figure formatting is inconsistent. Ensure uniform font size and style across all figures and verify that all axis labels and text elements are legible.**

**Response:We apologize for the inconsistency in figure formatting. We have redrawn Figure 3 and standardized its label format with other figures, added elements such as error lines, and provided the source files for all figures, though the final presentation may relate to typesetting.**

**7. The term “Derivative Index” is unclear. The authors should define this term explicitly when it is first introduced in the Results section.**

**Response:We agree that terms like “Derivative Index” and "Derived indicators" are vague and non-standard. We have replaced all such terms throughout the manuscript, especially in the Results section and Table 3 title, with more precise and standard descriptions: Derived Metrics. This is a standard term in machine learning and classification tasks, referring to metrics derived from the confusion matrix (e.g., PPV, TPR, etc.).**

**8. The manuscript contains numerous language and terminology issues. Ambiguous phrasing and redundant wording reduce clarity. Terminology should be corrected—for instance, "layer thickness" should be "slice thickness", "picture" should be "image", and "pieces" should be replaced with "instances", "images", or "patients" as appropriate. The manuscript also contains punctuation and grammatical errors that should be addressed through careful proofreading.**

**Response:We sincerely apologize for the language and terminology issues in the initial draft. We have undertaken a comprehensive language revision of the entire manuscript to improve clarity, fluency, and adherence to standard scientific English. Key terminology has been standardized (e.g., "images" instead of "pictures", "slice thickness" instead of "layer thickness", "samples" instead of "pieces"). We have also carefully corrected punctuation and grammatical errors throughout the text. We believe the manuscript has significantly improved in this regard.**

**9. The second line in the Introduction section seems unnecessary to the Introduction. Similarly, maternal disease is unclear; this could refer both to maternally inherited disorders or obstetric complications.**

**Response:We appreciate the reviewer's suggestion. We have removed the redundant phrase "commonly known as hyperlipidemia" from the second line of the Introduction. Regarding "maternal disease," we reviewed the cited literature and changed the term to the more precise "pregnancy-related disorders" to clarify the meaning.**

**10. In the Introduction, second paragraph, the wording is confusing. The reader could interpret HDL as “reversing cholesterol being transported to the liver” which implies that HDL transports cholesterol to the peripheral tissues. However, HDL is generally associated with "cholesterol transport to" the liver.**

**Response:We thank the reviewer for identifying this incorrect phrasing. We found the word "reverse" was erroneously inserted, leading to incorrect expression, so we have rewritten the sentence in the second paragraph of the Introduction to accurately describe the role of HDL-C.**

**We hope that our revisions and responses have adequately addressed the reviewers' concerns and improved the manuscript. We are committed to further enhancing the quality of our work and are open to any additional suggestions. Thank you once again for the opportunity to revise our manuscript. We look forward to your favorable consideration for publication.**

**Sincerely,**

**Junjiao Hu* (Corresponding author) and all co-authors**
